# Convergent and discriminant validity of the ImPACT with traditional neuropsychological measures

**DOI:** 10.1080/23311908.2018.1430199

**Published:** 2018-01-20

**Authors:** Robert J. Thoma, Julia A. Cook, Christopher McGrew, John H. King, Dalin T. Pulsipher, Ronald A. Yeo, Mollie A. Monnig, Andrew Mayer, Jessica Pommy, Richard A. Campbell

**Affiliations:** 1 Department of Psychiatry and Behavioral Sciences, University of New Mexico, Albuquerque, NM, USA.; 2 Department of Psychology, University of New Mexico, Albuquerque, NM, USA.; 3 Mind Research Network, Albuquerque, NM, USA.; 4 Division of Plastic & Reconstructive Surgery, Indiana University School of Medicine, Indianapolis, IN, USA.; 5 Department of Family and Community Medicine and Department of Orthopedics and Rehabilitation, University of New Mexico Health Sciences Center, Albuquerque, NM, USA.; 6 Akron Children’s Hospital, Neuro Developmental Sciences Center, Akron, OH, USA.; 7 Center for Alcohol & Addiction Studies, Brown University, Providence, RI, USA.

**Keywords:** Sports Injury, Neuropsychology, Testing, Measurement and Assessment, Sports Medicine, head injury, traumatic brain injury, concussion, test construction

## Abstract

Neuropsychological assessment of cognitive sequelae secondary to sports concussion is limited by lengthy administration times and lack of readily available neuropsychologists. Brief computerized test batteries are now under development to address this, but the validity of these measures is not yet established. The validity of one such computerized test battery, the Immediate Post-Concussion Assessment and Cognitive Testing (ImPACT), was administered to 93 healthy NCAA Division I athletes, aged 18–24, along with a battery of traditional, well-described neuropsychological tests. Convergent and discriminant validity between the ImPACT and traditional measures was investigated using multitrait-multimethod matrix (MTMM) analysis. As an example, the ImPACT Visual Motor Speed composite demonstrated reasonably good convergent validity secondary to moderate correlations with traditional measures of processing speed, but it demonstrated relatively poor discriminant validity as it significantly correlated with the Reaction Time composite score. MTMM results were variable across ImPACT composites and data for each are presented. The ImPACT composite’s validity was further investigated using exploratory factor analysis (EFA). Six principal components were termed processing speed, visual memory, verbal memory, attention & working memory, and verbal fluency, based upon traditional test loadings, and a sixth loaded only on the ImPACT Reaction Time composite. EFA indicated content validity of moderate strength for the Visual Motor Speed and Visual Memory composites, but revealed problems with specificity for the other composites. Based upon the present findings, validity problems render the interpretability of the ImPACT composites somewhat questionable, and more research is necessary prior to using the ImPACT for assessment of clinical populations.

## 1. Introduction

The summary from the Fifth International Conference on Concussion in Sport defines sports related concussion as “… a traumatic brain injury induced by biomechanical forces” (McCrory et al., 2017). Such a definition is inclusive but very imprecise, leading to substantial clinical heterogeneity. Due to this heterogeneity, concussion/mild traumatic brain injury (mTBI) in sports has received increasing attention in neuropsychology and sport medicine in recent years (Schatz, Pardini, Lovell, Collins, & Podell, 2006). Trainers and health care professionals working in athletic settings must diagnose and manage the 1.6–3.8 million sport-related concussions occurring in the United States annually (Broglio, Ferrara, Macciocchi, Baumgartner, & Elliott, 2007). Traditional neuropsychological tests are important diagnostic tools used by clinicians to assess neurocognitive impairment because neuropsychological tests may be sensitive to the subtle cognitive deficits associated with sports concussions in otherwise healthy, athletes ranging from elementary school age to the professional level (Collins, Lovell, & McKeag, 1999; Echemendia, Putukian, Mackin, Julian, & Shoss, 2001; Kelly, 2001; Macciocchi, Barth, Alves, Rimel, & Jane, 1996; McCrea, Kelly, Randolph, Cisler, & Berger, 2002; Randolph, McCrea, & Barr, 2005; Register-Mihalik et al., 2012).

A recent advance in neuropsychological sport concussion assessment has been the development of computerized neuropsychological tests, reflecting the need for easily implemented, portable testing for athletes who may be injured far from clinical support (Randolph et al., 2005). It has been proposed that traditional neuropsychological tests do not reveal the sometimes very mild decrements in functioning related to reaction time following a head trauma (Erlanger et al., 2003), but this remains to be determined. Computerized tests may be better suited to assess subtle changes in reaction time, with ease of administration and rapid scoring adding to the appeal of fully computerized neuropsychological measures (Broglio et al., 2007). However, the rapid dissemination and extensive use of computerized neuropsychological testing is of growing concern because the psychometric properties of these instruments have not yet been well characterized. Two additional concerns are that (1) athletic trainers who administer the computer assessments may lack both the background to effectively utilize and evaluate results of the computer-based tests; and (2) trainers’ access to a neuropsychologist as a consultant may be limited (Randolph et al., 2005).

Immediate Post-Concussion Assessment and Cognitive Testing (ImPACT, 2012; Lovell et al., 2006) is a computerized cognitive test battery that was designed specifically for the assessment of athletes prior to playing sports and after suffering a concussion. Administration time is roughly 20 min, and computer scoring provides composite scores for Verbal Memory, Visual Memory, Visual Motor Speed, Reaction Time, and Impulse Control (Schatz & Putz, 2006).

In two recent investigations, the validity of ImPACT was evaluated in relation to traditional neuropsychological measures. In the first (Iverson, Lovell, & Collins, 2005), modest-to-strong correlations for Verbal Memory (*r* = 0.37), Visual Memory (*r* = 0.46), and Reaction Time (*r* = 0.60) were found between ImPACT composite scores and the Symbol Digit Modalities Test, a traditional neuropsychological measure of information processing speed. Because this study only used one traditional neuropsychological measure, there is further need for independent investigation of the convergent and discriminant validity of computerized measures. In the second study (Maerlender et al., 2010), convergent validity was demonstrated for four of the five ImPACT composite scores, which were computed from a larger, comprehensive battery of traditional and experimental neuropsychological tests, including BVMT-R, California Verbal Learning Test, Word Memory Test, Continuous Performance Task, Pegboard, and Paced Auditory Serial Addition Test. Working memory, processing speed, and verbal working memory composite scores derived from traditional neuropsychological tests were moderately, but significantly, correlated with ImPACT Visual Motor and Reaction Time scores (correlations ranged from 0.34–0.41). The neuropsychological visual memory score was significantly correlated with ImPACT Verbal Memory (*r* = 0.44) and Visual Memory (*r* = 0.59), while the neuropsychological verbal memory composite was significantly correlated with ImPACT Verbal Memory (*r* = 0.40). There was no correspondence between traditional neuropsychological measures and ImPACT on impulse control scores, nor did the neuropsychological motor composite score correlate with any ImPACT measures. The present study also assessed convergent validity between traditional neuropsychological tests and ImPACT composite scores, but it also included an evaluation of discriminant validity, allowing for appraisal of the construct validity of ImPACT composite scores.

The present study was designed to assess the level of correspondence between well-understood traditional neuropsychological test results and those generated by the ImPACT test in a group of healthy university athletes (no current concussion). ImPACT was chosen for this comparison because it has emerged as one of the more popular and widely used computerized systems in post-concussion assessment (Guerriero, Proctor, Mannix, & Meehan, 2012). For example, of those practitioners who employ computerized measures for concussion assessment, 93% use ImPACT (Guerriero et al., 2012). It was predicted that ImPACT composite scores would significantly correlate with scores on traditional measures known to assess the same domains, demonstrating convergent validity. It was also hypothesized that ImPACT composite scores, purportedly representing independent cognitive domains, would not be significantly inter-correlated, showing discriminant validity. Additionally, we were interested in testing differences in neurocognitive performance between those with and without prior history of concussion.

## 2. Methods

### 2.1. Participants

Participants were 93 NCAA Division I student-athlete volunteers who played on the men’s football, men’s soccer, and women’s soccer teams. The sample consisted of 8 female and 85 male student-athletes between the ages of 18 and 24 (*M* = 20.31; *SD* = 1.33). Within this group, 60 participants reported no history of concussion, 21 reported a history of 1 prior concussion, 9 reported having 2 prior concussions, 2 reported having 3 prior concussions, and 1 reported a history of 6 prior concussions. Three of the players who had suffered recent prior concussions all reported that they were more than 80 days post-concussion, and the rest had suffered their most recent concussion more than a year prior to data collection. Those with a history of prior concussion were included if they had been either medically-cleared to return to play by the athletic department’s sports medicine physician or their concussions were sufficiently remote for it to be unlikely that they were still experiencing symptoms. Mean number of weeks since last concussion was 115.42 with a standard deviation of 102.65 (range = 11.43–313.71 weeks). Participants were screened by self-report for the presence of ADHD, seizures, and other neuropsychiatric disorders prior to study enrollment. All participants met the ImPACT-recommended validity criteria, an Impulse Control Composite Score greater than 20.

This research was approved by the University of New Mexico Human Subjects Research Review Committee (HRRC) and Institutional Review Board (IRB). Informed written consent was obtained for all participants.

### 2.2. Procedures

A battery of traditional neuropsychological tests was individually administered at the beginning of preseason in a quiet room by either a clinical neuropsychologist or a neuropsychological technician trained and supervised by a clinical neuropsychologist.

Computerized testing was conducted with a maximum of three athletes at a time in a small, quiet computer lab, proctored by a clinical neuropsychologist or post-doctoral fellow in neuropsychology. Traditional neuropsychological testing was immediately followed by ImPACT administration. Categories assessed by ImPACT include Visual Motor Processing Speed, Reaction Time, Impulse Control, Verbal Memory, and Visual Memory. Descriptions of the individual ImPACT tests can be seen in Table 1, while Table 2 shows the derivations of the ImPACT Composite scores using the ImPACT tests.

The battery of traditional neuropsychological tests was designed to assess cognitive domains that paralleled the five categories of composite scores calculated by the ImPACT. The traditional neuropsychological test battery required approximately 30 min to administer and included the following: (1) The Hopkins Verbal Learning Test-Revised (HVLT-R; Brandt & Benedict, 2001); (2) The Brief Visual Memory Test-Revised (BVMT-R; Benedict, 1997); (3) The Trail Making Test, Parts A & B (Trails; Reitan, 1958); (4) The Controlled Oral Word Association Test (COWAT; Borkowski, Benton, & Spreen, 1967); (5) The Wechsler Adult Intelligence Scale-IV (WAIS-IV; Wechsler, 2008) Symbol Search, Coding, and Digit Span Tests.

### 2.3. Validity and reliability of traditional neuropsychological measures

Neuropsychological testing is typically used selectively for sports-related concussion. For example, neuropsychological testing may be indicated when symptoms are prolonged or when there is a history of prior concussions. These tests are seen as such powerful tools because they have demonstrated reliability and validity. Test–retest reliability was confirmed between all six forms of the HVLT-R (Luciana, Conklin, Hooper, & Yarger, 2005; Macciocchi et al., 1996; Maerlender et al., 2010). Concurrent, convergent, and predictive validity were confirmed for this test by finding high correlations between HVLT-R and the immediate and delayed Logical Memory (*r* = 0.75, 0.77) and Visual Reproduction tests (*r* = 0.54, 0.69) of the Wechsler Memory Scale-Revised (Benedict, Schretlen, Groninger, & Brandt, 1998; Robbins, 2008; Shapiro, Benedict, Schretlen, & Brandt, 1999). In contrast, Lacritz and Cullum (1998) demonstrated more modest correlational values between HVLT-R and CVLT in a trial of 25 elderly participants: the first learning trial showed a correlation of *r* = 0.30; the second trial had a correlation of *r* = 0.31; and the third and fourth trials displayed correlations of *r* = 0.65. The BVMT-R also has demonstrated reliability and validity. The BVMT-R has high correlations with other tests of verbal and visual memory, including the HVLT-R Total (*r* = 0.73) and Delayed Recall (*r* = 0.74) tests. It was also found to be highly correlated with the Visual Reproduction subtests of the Wechsler Memory Scale-Revised; specifically, the learning trials (*r* = 0.66) and delayed recall tests (*r* = 0.80; Robbins, 2008). Trails A has a reported interrater reliability of 0.94, and its coefficient of concordance was found to be 0.98 (Lezak, 2004; Robbins, 2008). Trails A was also shown to differentiate between individuals with and without brain damage (Reitan, 1955, 1958; Robbins, 2008). Trails B has a reported coefficient of concordance of 0.90 (Fals-Stewart, 1992; Lezak, 2004) and a one-year retest reliability of 0.72 (Robbins, 2008; Snow, Macartney-Filgate, Schwartz, Klonoff, & Ridgley, 1988). COWAT has been shown to be able to differentiate between Alzheimer’s patients, patients with mild traumatic brain injuries, and control participants (Raskin & Rearick, 1996; Robbins, 2008). Finally, Coding had an average stability coefficient of 0.83, and the average stability coefficient of Symbol Search was 0.79 (Robbins, 2008; Wechsler, 2008).

### 2.4. Statistical analyses

To determine convergent and discriminant validity, we created a multitrait-multimethod matrix (MTMM) by examining partial correlations (using age as covariate) between ImPACT domain scores and scores from traditional neuropsychological measures. Age was entered as a covariate in all analyses because neurodevelopmental changes continue to occur into early adulthood (Luciana et al., 2005) and age effects, even if non-significant, might bias any cognitive findings. To minimize the possibility of introducing bias due to heterogeneity across published normative samples, raw test scores were used to compute partial correlations, using age as a covariate. The MTMM is an approach designed by Campbell and Fiske (1959) to enhance the identification of convergent and discriminant validity of the measurements by tabulating the correlations between tests. Convergent validity refers to the degree of relatedness between tests that should be similar. Discriminant validity, in contrast, refers to the extent to which a test can differentiate between unrelated traits (Hayashi, 1987). Monomethod-monotrait correlations examine the relationship between scores thought to assess the same construct using the same method. In other words, this is the correlation of the measure with itself and should equal 1.0. Monotrait-heteromethod correlations report the relationship between different measures used to assess the same general construct.

Correlations would be expected to be high if ImPACT assesses the same construct as traditional neuropsychological measures, thereby demonstrating convergent validity. Heterotrait-heteromethod correlations explore the relationship between different measures assessing different constructs. Therefore, correlations would be expected to be low, providing evidence of discriminant validity.

ImPACT domains and traditional neuropsychological measures were grouped into one of five categories based on common practice: Processing Speed, Executive Functions, Learning, Verbal Memory, and Visual Memory. We examined ImPACT domain scores rather than individual ImPACT subtest scores to reduce the overall number of comparisons and because domain scores are most commonly referenced in clinical practice. Rather than create artificial domain scores from the traditional neuropsychology measures, we examined individual subtests. In clinical practice, clinicians rarely create their own domain scores from different measures that have different normative samples.

Exploratory factor analysis (EFA) was used to investigate the factor structure underlying the test battery presented in the MTMM (Table 3) and to further explore the content validity of the ImPACT composite scores in terms of traditional neuropsychological measures. While there were a priori expectations regarding which tests should show strong convergent and discriminant validity, there were no hypotheses rendered regarding the underlying factor structure. Hence, EFA was implemented using principal component analysis (PCA) in SPSS and VARIMAX rotation was applied to maximize the interpretability of the resulting pattern of component loadings (Hill & Hughes, 2007).

## 3. Results

### 3.1. Multitrait-multimethod matrix

Table 3 shows the partial correlations between ImPACT Composite Scores and all traditional neuropsychological test scores organized in terms of an MTMM. Since sample size plays such a large role in statistical significance, we compared correlations relative to other tests, rather than using significance as a determination of construct validity. Our correlations were most consistent with those modest correlations found by Lacritz and Cullum (1998), which ranged from *r* = 0.30–0.65 for learning trial correlations between HVLT-R and CVLT.

#### 3.1.1. Processing speed

With regard to convergent validity within the Processing Speed domain, three traditional neuropsychological measures, Symbol Search (*r* = 0.39, *p* < 0.01) and Coding (*r* = 0.40, *p* < 0.01) and Trails A (*r* = 0.33, *p* < 0.01) were significantly correlated with ImPACT Visual Motor composite score. Coding (*r* = −0.25, *p* < 0.05) and Trail Making Test, Part A (*r* = −0.36, *p* < 0.01) were significantly correlated with ImPACT Reaction Time. There was a number of significant correlations between ImPACT Visual Motor and other tests in the four other domains that would not be expected (e.g. with several memory scores), suggesting a lack of discriminant validity. Further evidence for poor discriminant validity were the correlations found between the Visual Motor Composite score and other ImPACT Composite scores. For example, the Visual Motor Composite was significantly correlated with the Verbal Memory composite score (*r* = 0.22; *p* < 0.05) and showed trend level correlations with the Verbal Memory composite score (*r* = 0.19, *p* = 0.09), and the Impulse Control composite score (*r* = −0.20, *p* = 0.07). Symbol Search, a speeded test that also involves working memory, was also correlated with seven non-processing speed scores, although most of those measures were also speeded and/or required working memory.

#### 3.1.2. Executive functions

The only significant correlation between ImPACT Impulse Control and traditional measures was with COWAT Animals (*r* = 0.22, *p* < 0.05). No other executive function measures were related to Impulse Control, but there were several significant relationships between neuropsychological test scores both within the Executive Function domain and in other domains. In particular, BVMT-R Delayed Recall scores were significantly correlated with traditional measures of executive functioning.

#### 3.1.3. Verbal memory

Both HVLT-R memory scores (Delayed Free Recall and Percent Retention) had moderate, but statistically significant correlations with the ImPACT Verbal Memory and Visual Motor Speed composite scores.

#### 3.1.4. Visual memory

In contrast to verbal memory, neither BVMT-R Delayed Free Recall, nor Recognition scores significantly correlated with ImPACT Visual Memory. Notably, the correlations between BVMT-R Delayed Free Recall (*r* = 0.16, ns) and Recognition (*r*=−0.11, ns) and ImPACT Visual Memory score were weak, which was unexpected.

### 3.2. Other correlations

#### 3.2.1. ImPACT

Intercorrelations between the ImPACT Composite Scores are also summarized in Table 3. The ImPACT Visual Motor Speed composite was significantly correlated with ImPACT Verbal Memory and Reaction Time composites, but correlations were small, suggesting good discriminant validity among ImPACT composite scores.

### 3.3. Exploratory factor analysis

Exploratory factor analysis (EFA) was used to explore the component structure underlying the test battery presented in Table 3. PCA with VARIMAX rotation revealed six underlying components with eigenvalues greater than 1. Those six components accounted for 66.02% of the total variance and the percent of variance accounted for ranged from 16.85% for Component 1 to 7.07% for Component 6. Table 4 shows the component loadings for each test. Component names were derived by the authors based upon the patterns of factor loadings for each.

Of the ImPACT Composites, Component 1 has the strongest loadings on the Visual Motor Speed composite score (0.682) and the Reaction Time Composite score (−0.527), suggesting that Component 1 may be best defined as a measure of visuomotor speed, with the emphasis on speed. The finding that Component 1 also had strong loadings on Trails A (−0.742), Trails B (−0.792), WAIS-IV Coding (0.707), and WAIS-IV Symbol Search (0.590), all traditional neuropsychological measures that include a processing speed component, provides additional support for Component 1 as a measure of processing speed. Together, these findings suggest that the ImPACT Visual Motor Speed composite score has relatively strong content validity, and lend support to the findings of the multitrait-multimethod analysis of this score having relatively good convergent validity. The high loading of the ImPACT Reaction Time Composite score on Component 1, however, is consistent with multitrait-multimethod results showing relatively poor discriminative validity within the ImPACT battery.

Component 2 loaded most highly on the ImPACT Visual (0.559) and Verbal (0.480) Memory composite scores, suggesting that this component may represent a general memory component. However, the pattern of loadings for Component 2 on traditional neuropsychological test scores strongly suggests that Component 2 is specific to visuospatial memory. For example, Component 2 loadings for BVMT-R Total Recall score (0.747) and Delayed Recall score (0.737) were quite strong, whereas loadings for HVLT-R Total Recall score (0.013) and Delayed Recall score (0.115) were minimal. The disagreement in the pattern of loadings between the ImPACT memory composites and traditional neuropsychological memory tests is suggestive of relatively low content validity of one or both of the ImPACT memory composites and consistent with multitrait-multimethod results showing relatively poor discriminative validity within the ImPACT battery.

Component 3 appears to be specific to verbal memory due to its high loadings on HVLT-R Total Recall (0.823) and Delayed Recall (0.850) scores, and minimal loadings on BVMT scores. In agreement with this notion, the ImPACT Verbal Memory composite score has a moderate loading on Component 3 (0.497) and the ImPACT Visual Memory composite score loading is minimal (−0.102). Combined with the pattern of loadings for Component 2, this result suggests that the ImPACT Verbal Memory composite has moderate content validity with regard to the verbal memory domain, but the lack of specificity indicates problems with divergent validity.

Component 4 has a moderate positive loading on the ImPACT Reaction Time score (0.409) and loadings of minimal strength on all other ImPACT composites, suggesting relatively good discriminative validity. It is not possible to assess content validity in terms of the extent to which the ImPACT Reaction Time composite score samples the domain of reaction time since there were no tests of reaction time in the battery of traditional neuropsychological measures. However, Component 4’s strong loadings on tests associated with memory span capacity (Digits Forward [0.833]) and working memory (Digits Backward [0.730]; Digit Sequencing [0.458]) suggests that it has poor specificity as a test of pure reaction time and might be better thought of as a measure of attention and working memory.

Based upon its strong loadings on the COWAT FAS (0.840) and Animals (0.684) tests and minimal loadings across all other tests, Component 5 may be best considered as a measure of general verbal fluency. As problems with verbal fluency are an indicator of post-concussion injury to fronto-temporal cortex and have predictive value regarding the course of cognitive recovery, an inability to measure verbal fluency represents a major weakness of the ImPACT which is endemic to all computerized test batteries designed to assess post-concussion cognitive functioning.

The ImPACT Impulse Control composite score loads strongly on Component 6, and appears to have relatively good specificity, as there are only weak loadings across almost all other neuropsychological tests. A moderate positive loading with the COWAT Animals score suggests that Component 6 may also sample the domain of executive functioning. This finding also serves to again point out a weakness in our selection of tests for the traditional neuropsychological test battery in that we did not include any measures of validity.

### 3.4. Effect for remote history of concussion

To determine whether previously concussed athletes differed from athletes who never had experienced a concussion, we conducted a one-way ANOVA comparing athletes with a history of at least one concussion (*n* = 33) and had been subsequently cleared to play after making a full recovery, to athletes who had never suffered a concussion (*n* = 60). There were no significant differences on either ImPACT composite scores or on any traditional neuropsychological test scores between the groups (see Table 5).

## 4. Discussion

In the literature on sports concussion, reaction time (RT), information processing, memory, attention, and executive functioning are commonly reported neuropsychological symptom domains affected by concussion (Collie, Darby, & Maruff, 2001; Harrington, 2008). Unfortunately, little is known about the correspondence between traditional neuropsychological testing and computerized measures such as the ImPACT with regard to assessment of these domains. To address this issue, the present study was designed to assess the validity of the ImPACT by means of comparison to traditional clinical tests for which the validity has already been established through extensive clinical use and experimental research. Hence, the HVLT-R, BVMT-R, Trail Making Test, COWAT, and subtests of the WAIS-IV were selected for comparison purposes. Since construct validity of a measure is established when both convergent and discriminant validity are independently demonstrated, with respect to comparison measures, our first objective was to establish convergent validity between the ImPACT composite scores and traditional neuropsychological test scores. To do so, the multitrait-multimethod matrix (MTMM) method was employed as an efficient means of rendering convergent and discriminant features immediately discernible (Campbell & Fiske, 1959). To consider content validity in terms of the underlying factor structure of this battery of tests, exploratory factor analysis (EFA) using principal components analysis (PCA) was also employed. Lastly, all tests in the battery were evaluated with regard to their ability to detect remote history of concussion using one-way analysis of variance.

The current results suggest relatively strong construct validity for the ImPACT Visual Motor Speed Composite score as a measure of processing speed. As can be seen in Table 3, the ImPACT Visual Motor Speed composite score was significantly correlated with the WAIS-IV Symbol Search and Coding scores, subtests that may readily be construed as measures of visual motor processing speed. The Visual Motor score also correlated with the Trail-Making Test, Parts A and B, both of which include a psychomotor processing speed component. Hence, the ImPACT Visual Motor Speed composite score was moderately positively correlated with all four traditional measures of processing speed associated with visual stimuli. The EFA revealed that the Impact Visual Motor Speed composite score had a strong loading on the first principal component, which was defined by strong loadings with traditional measures assessing processing speed (see Table 4). In addition to visual motor response speed, however, the ImPACT Visual Motor Speed composite score is described as evaluating learning and memory and visual processing (ImPACT Technical Manual, 2012; see also Table 1). With respect to its convergence with traditional measures of visual memory and learning, in the MTMM, weak but statistically significant correlations with BVMT-R Total and Delayed Recall scores lend limited support for convergent validity in this domain. With regard to the specificity of the ImPACT Visual Motor Speed composite score, MTMM revealed significant correlations with tests in the Executive Function domain, WAIS-IV Digit Span Backward and Trails B; a finding suggesting that as broadly as the content of this composite is described, it may also sample executive functioning, suggesting questionable discriminant validity. It is also notable that although the descriptor, “learning and memory” in the test explanation was not limited to tests of visual learning and memory, the Visual Motor Speed Composite score was also unexpectedly significantly correlated with the HVLT-R Delayed Recall score. Regarding discriminant validity with respect to other ImPACT composite scores, the Visual Motor Speed composite score was significantly correlated with the Reaction Time and Verbal Memory composite scores in the MTMM, indicating a relative inability to discriminate between any tests containing a speed element. The significant negative correlation found with the ImPACT Impulse Control composite score may reflect poor discriminant validity, or perhaps might indicate the presence of a speed-accuracy trade-off, which could be construed as an unexpected advantage of the ImPACT. Future research will be necessary to address that possibility. PCA revealed questionable content validity for the Visual Motor Speed composite, in that both the Visual Motor Speed and Reaction Time composite scores strongly loaded on the processing speed component (Table 4, Component 1). In review, the ImPACT Visual Motor Speed composite score evinced good convergent validity with traditional tests within the boundaries of its defined neurocognitive domains. There was evidence suggesting reasonable discriminant validity, in that it was not significantly correlated with seven of nine traditional test scores that might be considered outside its defined domain, but it was significant that three of the four other ImPACT composite scores indicated the presence of problems with discriminant validity within the ImPACT battery, rendering this composite of questionable clinical value.

A second ImPACT composite score with reasonable convergent validity with respect to traditional neuropsychological test scores was the Verbal Memory Composite score. It is defined in the ImPACT Manual as evaluating memory, learning and attentional processes within the verbal domain (ImPACT Technical Manual, 2012). With respect to convergent validity with traditional verbal memory scores, in the MTMM, the Verbal Memory composite evinced moderate-sized, statistically significant, positive correlations with HVLT-R Delayed Memory, and HVLT-R Percent Retention scores. In the EFA, the Verbal Memory composite score had a moderate loading on Component 3; a component with a strong loading on the HVLT-R Total Recall and Delayed Recall subtests. All of these findings indicate relatively strong convergent validity for the Verbal Memory Composite score with tests of verbal memory. Further analysis of the MTMM findings revealed a significant positive correlation between the ImPACT Visual and Verbal Memory composites, and examination of the EFA results indicated that the Verbal Memory composite had a loading of moderate strength on the visual memory component (Component 2). These findings indicate that the ImPACT Visual Memory composite score has poor discriminant validity between memory domains, which renders the content validity somewhat questionable due to a lack of specificity. Evaluation of the ImPACT Verbal Memory composite score with regard to traditional tests of attention revealed only a weak, but significant correlation with a single traditional measure of attention, the Digit Span Sequencing score, and non-significant correlations with the Digit Span Forward and Backward scores; findings demonstrating poor convergent validity with tests of attention and inconsistency with the notion that the Verbal Memory composite score might be used as a measure of attention. It is notable though, that whereas the ImPACT Verbal Memory subtests are administered visually on a computer screen, the WAIS-IV tests of attention are administered aurally, and future research is needed to determine whether the present findings of poor convergent validity in the attention domain may be due to the sensory modality in which stimuli are presented. Regarding the Verbal Memory composite score’s convergent validity with other measures of verbal learning, in the MTMM, the Verbal Memory composite score was found to be unrelated to the HVLT-R Total Recall score, but moderately significantly correlated to the BVMT-R Total Recall score, indicating both poor convergent and discriminative validity with respect to traditional tests of the respective learning domains. In review, the ImPACT Verbal Memory composite score was uncorrelated with all six of the traditional neuropsychological scores to which it should not be related in the MTMM. With regard to discriminant validity within the ImPACT domain scores, it was perhaps appropriately, significantly correlated with the ImPACT Visual Motor Speed composite. However, it was also significantly correlated with the ImPACT Visual Memory Composite, indicating poor discriminative validity within the memory domain and questionable construct validity as a test specific to verbal memory. Further, the ImPACT Verbal Memory Composite score demonstrated poor validity as a measure of attention and verbal learning.

The ImPACT Visual Memory composite score is purported to measure “visual processing, learning and memory, and visual motor response speed.” However, MTMM analysis showed that it was only weakly correlated with the BVMT-R Total Recall score, a measure of visual learning, and was unrelated to visual memory measures, the BVMT-R Delayed Recall and Recognition subtests. It was also unrelated to any measures of visual processing or visual motor response speed. This pattern suggests convergent validity with visual learning, but not with visual memory (or any of the other cognitive skills described in the definition). With regard to discriminant validity with traditional neuropsychological tests, it was weakly correlated only with the WAIS-IV Digit Span Backward score. Within the set of ImPACT composite scores, it was significantly correlated with the ImPACT Verbal Memory composite score suggesting possible problems with discriminant validity. In the EFA, The Visual Memory Composite score evinced a moderate loading on Component C2, which had strong loadings with the BVMT learning and memory scores and had small to moderate loadings with the Digit Span measures of working memory. Concurrent MTMM findings of poor convergent and poor discriminative validity suggest that the Visual Memory composite has little content validity and that it is perhaps more closely related to working memory, perhaps allowing one to temporarily store visual stimuli in a short-term visual memory buffer and resulting in a high correlation with visual learning. However, it is not specifically related to one’s ability to remember and recall or recognize visual information. Hence, the name, “Visual Memory” may misrepresent this particular composite.

The ImPACT test battery includes a composite score specifically dedicated to the assessment of reaction time, the ImPACT Reaction Time composite score. As measures of reaction time can be a relatively direct means of measuring attention deficits (Lezak, 2004), this is perhaps a relative strength of the ImPACT battery. There are few validated traditional neuropsychological measures of reaction time for clinical use, and none were included in the present traditional testing battery due to time constraints and as a result it was not possible to evaluate the convergent validity of this test. As the Reaction Time composite is defined as “average response speed” (ImPACT Technical Manual, 2012), it is notable that it was negatively correlated with Trails B of the traditional tests, perhaps reflecting a strategy of speed-accuracy trade-off on Trails B. Similarly, the Reaction Time composite was weakly negatively correlated with the ImPACT Visual Motor Speed composite, perhaps reflecting the same strategy. However, as previously noted, in the EFA, the Reaction Time composite score evinced a moderate loading on a component that was most strongly related to traditional tests of processing speed, and strongly loaded on the ImPACT Visual Motor Speed component, adding further doubt to the relative discriminant validity, and hence the content validity, of the Impact Reaction Time composite score.

The Impulse Control composite score is defined as providing a measure of errors on testing, useful in determining test validity, but not for clinical decision-making. This suggests that the Impulse Control composite may be of some use in interpretation of the other composite scores but should not be included as a Cognitive Domain score, as one might be led to believe by its inclusion among the other neurocognitive domain scores. Consistent with the notion of the Impulse Control composite as an entirely independent measure, the EFA results indicated that it loaded on a component that did not have more than weak loadings on any other test score (Component 6). This finding serves to point out a weakness of the present study design, in that no validity measures were included in the traditional neuropsychological test battery, and a future direction for research might be to investigate how it related with other measures of test validity and effort.

The present analyses also revealed substantial problems in the convergent and discriminant validity among the ImPACT composite scores, indicating poor construct validity of all but the ImPACT Visual Motor Speed composite scores. Maerlender et al. (2010) reported that convergent validity was demonstrated for four of the five ImPACT composite scores, which were correlated with composites compiled from a series of traditional neuropsychological tests. The authors reported moderate correlations between the relevant traditional composite scores and ImPACT Visual Motor Speed, Verbal Memory, and Reaction Time, scores; with particularly high correlations between Visual Memory measures. The present research is consistent with Maerlender et al. (2010) in reporting reasonably good convergent validity for the ImPACT Visual Motor Speed and Verbal Memory composite scores. However, there is disagreement regarding the Visual Memory composite score, which demonstrated poor convergent validity. Iverson et al. (2005) found that when compared to the Symbol Digit Modalities Test (SDMT), there were modest correlations with ImPACT Visual Memory composite score (*r* = 0.46, *p* < 0.01) and ImPACT Verbal Memory composite score (*r* = 0.37, *p* < 0.01). Additionally, those authors found strong correlations between SDMT and ImPACT Reaction Time composite score (*r* = −0.60, *p* < 0.01). While our results do replicate the modest correlation between the ImPACT Visual and Verbal Memory composite scores, we were not able to replicate their modest-to-strong correlations between traditional neuropsychological tests and the ImPACT Visual Memory or ImPACT Reaction Time composites. The present results show some interesting similarities, however, with those reported by Schatz and Putz (2006). Both studies found significant, but perhaps unlikely correlations between traditional tests Trails B and Digit Span Backward and the ImPACT Visual Motor Speed Composite score.

Our results suggest that the ImPACT has relative strengths and weaknesses as a tool for measuring cognitive skills. Strengths include easy implementation, the inclusion of reaction time measures (Erlanger et al., 2003), the Visual Motor Speed Composite’s relatively strong construct validity (Erlanger et al., 2003), and the relatively strong convergent validity (but weak discriminant and construct validity) of the Verbal Memory composite score. All three of these domains can be sensitive indicators of brain injury. The weaknesses include the lack of construct validity found for the ImPACT Visual Memory composite score and the inclusion of the non-construct-related, and potentially misleading, Impulse Control composite score.

What might account for the discontinuities found between the ImPACT and this set of traditional measures? A first possibility is that administration environments were slightly different for each of these measures. While the traditional neuropsychological tests were administered in a small testing room with only the test administrator and subject involved, the administration of the ImPACT was in a small, quiet computer lab with cubicles allowing up to four participants taking the test at the same time. Although participants were supervised by an experimenter at all times during the ImPACT session, it may be that variables such as the group format or examiner presence resulted in biased test performances. It seems unlikely that environmental noise would be so disruptive, however, since the ImPACT was designed to be administered either individually or by group. A second possible source of bias may be that whereas the ImPACT was administered precisely the same way (via computer) to each subject, the traditional neuropsychological tests were administered and scored by different researchers. However, this possibility is also considered dubious, as all researchers were trained to competence, and all test scoring was done blind to participant identity. A third potential source of discrepancy between test batteries is that while differences in sensory modalities are known to have no significant difference on memory (i.e. verbal versus auditory administration; Collie et al., 2001), each of the ImPACT tests requires a motor component, which is not required in the HVLT-R Total Recall, HVLT-R Delayed Recall, HVLT-R Recognition, or BVMT-R Recognition. According to Topolinski (2012), there exists a crucial interaction between the memory component of their study and non-related motor interference, such that the inclusion of a motor component serves to strengthen implicit memory and familiarity. A fifth source of divergence between the batteries is the lack of executive function measures in the ImPACT. Deficits in executive functions, such as organization, planning, cognitive flexibility, inhibition control, problem solving, and working memory, and impulse control may all be indicative of the presence of mild TBI (Topolinski, 2012). The lack of an executive measure of any kind renders the ImPACT a less sensitive test than it might otherwise be (Brooks, Fos, Greve, & Hammond, 1999).

As with any study, there are limitations associated with the present study. One limitation is our relatively small sample size, resulting in a population of athletes that was fairly homogeneous in terms of the sports that were represented. Small sample size also rendered us unable to adequately address individual differences that may have affected the results, such as age and gender effects. Our inability to address the issue of potential gender differences due to the small sample of female athletes (*N* = 8) is unfortunate, but may reflect the population that was sampled. In follow-up studies, a greater effort will be make to address the important issue of gender effects on test validity. Also, more comprehensive traditional neuropsychological battery would provide more information about more subtle sources of content discrepancies than was possible with the brief battery we selected for time efficiency. A potential weakness of the present study was a lack of counterbalanced order of administration between the two test batteries and future studies would also be strengthened by more carefully controlling for possible order effects. Lastly, although there was no mean difference in the present sample between athletes with a history of concussion and those without, a future direction for this research might involve assessment of test validity in samples with remote history of concussion or in those with acute concussion symptoms.

In conclusion, the ImPACT has distinct strengths and weaknesses with regard to the sample of cognitive abilities it purports to assess (Nelson et al., 2016) and more research is necessary to conclusively establish the validity of the battery. On the other hand, the ImPACT represents an important step in the advancement of computerized testing devised to improve the safety of athletes.

## Figures and Tables

**Table 1.** ImPACT subtest descriptions

ImPACT subtest	Brief description
Word Memory	Learn 24 semantically similar target and non-target words, then indicate whether presented word is target word
Symbol Match	Remember symbol number pairings and match number to given symbol
Three Letter Memory	Distractor: Click numbers 1–25 in backward order
	Test: Recall three letters presented before distractor
Design Memory	Learn 24 target and non-target designs, then indicate if presented design is target or non-target
X’s and O’s	Distractor: Press “Q” for blue square and “P” for red circle
	Test; then indicate location of highlighted X’s and O’s presented before distractor
Color Match	Determine whether color-related word is written in same color ink as the color the word describes

**Table 2.** Derivation of ImPACT composite scores

Composite score	Targeted cognitive functions	Derivation of score
Visual Motor Speed	Visual processing, learning and memory, and visual motor response speed	Computed as the average of the *Total Number Correct/4 during Interference of X’s and O’s* and the *Average Counted Correctly from the Countdown Phase of Three Letters* scores
Reaction Time	Average response speed	Computed as the average of the *Average Correct RT of Interference Stage of X’s and O’s, Symbol Match Average Correct RT Visible/3*, and the *Color Match Average Correct RT* scores
Impulse Control	A measure of errors on testing and for determination of test validity	Computed as the sum of the *Total Incorrect on the Interference Phase of X’s and O’s* and *Color Match Total Commissions* scores
Verbal Memory	Attention, learning, and memory within the verbal domain	Computed as the average of the Word Memory Total Percent Correct, Symbol Match [(Total Correct Hidden)/9*100], and *Three Letters Percent Total Letters Correct* scores
Visual Memory	Visual attention and scanning, learning, and memory	Computed as the average of the *Design Memory Total Percent Correct* and X’s and O’s [(Total Correct Memory/12*100] scores

**Table 3.** Multitrait-multimethod matrix of ImPACT composite scores and traditional neuropsychological tests (Raw scores)

			Processing Speed					Executive Functions							Learning		Verbal Memory				Visual Memory	
			ImPACT Visual Motor Speed	Symbol Search	Coding	ImPACT Reaction Time	Trails A	ImPACT Impulse Control	Animals	FAS	Trails B	Digit Span			HVLT-R Total Recall	BVMT -R Total Recall	ImPACT Verbal Memory	HVLT-R Delayed Recall	HVLT-R % Retention	ImPACT Verbal Memory	HVLT-R Delayed Recall	HVLT-R % Retention
												Sequencing	Backward	Forward								
Processing Speed	ImPACT Visual Motor Speed		1.00																			
	Symbol Search		0.39**	1.00																		
	Coding		0.40**	0.31**	1.00																	
	ImPACT Reaction Time		−0.27**	−0.05	−0.25*	1.00																
	Trails A		^0.33^**	0.34**	.28*	−0.15	1.00															
Executive Functions	ImPACT Impulse Control		−0.20*	0.11	−0.08	0.07	0.02	1.00														
	Animals		0.18	0.30**	0.18	0.06	0.33**	0.22*	1.00													
	FAS		0.13	0.34*	−0.03	−0.04	−0.19	−0.12	0.34**	1.00												
	Trails B		0.41**	0.38**	0.35**	−0.27**	0.53**	0.11	0.19	0.06	1.00											
	Digit Span	Sequencing	0.19	0.23*	0.05	−0.13	0.21*	−0.04	0.06	0.16	0.30**	1.00										
		Backward	0.27**	0.24*	0.05	0.08	0.09	−0.03	0.15	0.30**	0.23*	0.39**	1.00									
		Forward	0.02	0.15	−0.12	0.20	0.05	0.00	−0.02	0.19	0.10	0.29**	0.43**	1.00								
Learning	HVLT-R Total Recall		0.19	0.18	0.18	0.07	0.05	−0.01	0.23*	0.06	0.05	0.13	0.15	0.00	1.00							
	BVMT-R Total Recall		0.29**	0.25*	0.07	−0.03	0.18	0.04	0.27**	0.20	0.12	0.37**	0.35**	0.19	0.23*	1.00						
Verbal Memory	ImPACT Verbal Memory		0.22*	−0.08	0.05	0.14	0.07	−0.16	0.13	0.11	0.06	0.20*	0.06	0.08	0.18	0.36**	1.00					
	HVLT-R Delayed Recall		0.24*	0.21*	0.19	0.01	0.08	−0.09	0.12	0.09	0.05	0.14	0.12	0.06	0.60**	0.23*	0.35**	1.00				
	HVLT-R % Retention		0.19	0.06	0.17	−0.08	−0.05	−0.09	0.08	0.04	0.02	0.11	−0.01	0.01	0.16	0.16	0.33**	0.71**	1.00			
Visual Memory	ImPACT Visual Memory		0.19	0.11	−0.03	−0.08	0.07	−0.01	−0.06	0.20	0.10	0.17	0.28**	0.09	0.09	0.28**	0.20*	0.11	0.13	1.00		
	BVMT-R Delayed Recall		0.31**	0.18	0.13	−0.12	0.22*	0.03	0.21*	0.09	0.24*	0.37**	0.29*	0.20	0.12	0.61**	0.23*	0.22*	0.16	0.16	1.00	
	BVMT-R Recognition		0.14	0.07	−0.08	−0.01	0.32**	−0.04	0.00	0.01	0.03	0.01	0.04	0.20*	−0.08	0.37**	0.00	−0.03	−0.11	0.02	0.14	1.00

Notes: Yellow = Significant Monotrait-Monomethod correlations; Red = Significant Monotrait-Heteromethod correlations; Light Blue = Nonsignificant Heterotrait-Heteromethod correlations; Darker Blue = Significant Heterotrait-Heteromethod correlations that are outside of the above four categories but can be seen as correlations share cognitive properties; Green = Unpredicted significant correlations

* *p*<0.05.

** *p*<0.01.

**Table 4.** Principal component analysis factor loadings*

Test Name**	Component					
	1. Processing speed	2. Visual Memory	3. Verbal Memory	4. Attn & Working Memory	5. Verbal Fluency	6. Impulse Control
ImPACT Verbal Memory	−0.102	0.480	0.497	0.030	0.084	−0.205
ImPACT Visual Memory	−0.023	0.559	−0.102	0.003	0.178	−0.186
ImPACT Visual Motor Speed	0.682	0.222	0.203	0.100	0.036	−0.263
ImPACT Reaction Time	−0.527	−0.334	0.234	0.409	0.156	0.201
ImPACT Impulse Control	−0.022	0.009	−0.085	−0.037	0.025	0.865
HVLT-R Total Recall	0.084	0.013	0.823	0.000	0.118	0.006
HVLT-R Delayed Recall	0.120	0.115	0.850	−0.023	−0.026	−0.020
BVMT-R Total Recall	0.142	0.747	0.263	0.263	0.115	0.200
BVMT-R Delayed Recall	0.155	0.737	0.192	0.267	−0.095	0.180
Trails A (seconds)	−0.742	−0.036	0.058	0.005	−0.262	−0.122
Trails B (seconds)	−0.792	−0.238	0.015	−0.141	−0.049	−0.118
WAIS-IV Digit Span Back	0.164	0.195	−0.007	0.730	0.187	−0.128
WAIS-IV Digit Span Seq	0.309	0.399	0.011	0.458	0.031	−0.072
WAIS-IV Digit Span Forward	−0.100	0.118	−0.039	0.833	−0.014	0.063
WAIS-IV Coding	0.707	−0.201	0.214	−0.092	−0.075	−0.084
WAIS-IV Symbol Search	0.590	−0.084	0.094	0.273	0.404	0.199
COWAT FAS	0.049	0.174	−0.018	0.150	0.840	−0.184
COWAT Animals	0.226	0.049	0.232	−0.011	0.684	0.362

* Extraction Method: Principal Component Analysis; Rotation Method: Varimax with Kaiser Normalization; a Rotation converged in 10 iterations.

** HVLT-R % Retention and BVMT-R Recognition scores were not entered into the PCA due to the extent of shared variance with other memory measures.

**Table 5.** Analysis of remote history of concussion

Test Name	Concussion history*	M(SD)**	*F*-statistic	*p*-value
ImPACT Verbal Memory	−	86.26(10.87)	0.49	0.49
	+	87.63 (8.47)		
ImPACT Visual Memory	−	75.40 (12.42)	0.59	0.44
	+	73.61(10.53)		
ImPACT Visual Motor	−	39.86 (5.87)	0.35	0.55
	+	39.14 (6.55)		
ImPACT Reaction Time	−	0.60(0.08)	0.23	0.63
	+	0.59(0.07)		
ImPACT Impulse Control	−	5.16(3.47)	1.34	0.24
	+	4.39(3.18)		
WAIS-IV Symbol Search	−	54.1(8.2)	0.31	0.58
	+	53.0(7.3)		
WAIS-IV Coding	−	51.9(7.2)	0.70	0.41
	+	50.5(6.2)		
WAIS-IV Digit Span Forward	−	48.4(7.5)	0.48	0.49
	+	47.1(9.6)		
WAIS-IV Digit Span Backward	−	49.5(8.1)	0.46	0.50
	+	48.2(7.8)		
WAIS-IV Digit Span Sequence	−	50.6(8.8)	1.71	0.19
	+	53.1(7.3)		
FAS	−	43.5(14.0)	0.22	0.64
	+	42.2(14.1)		
Animals	−	50.1(9.3)	0.29	0.59
	+	49.1(0.93)		
Trails A	−	52.5(7.7)	1.15	0.29
	+	54.3(7.8)		
Trails B	−	48.5(12.9)	0.69	0.41
	+	50.7(11.1)		
HVLT-R Total Recall	−	43.7(10.2)	0.97	0.42
	+	41.5(10.6)		
HVLT-R Delayed Recall	−	44.71(10.6)	1.52	0.22
	+	41.88(11.1)		
HVLT-R Retention %	−	47.8(12.8)	0.68	0.41
	+	45.5(12.8)		
BVMT-R Total Recall	−	46.3(11.7)	0.32	0.57
	+	44.8(14.5)		
BVMT-R Delayed Recall	−	46.1(12.9)	0.29	0.59
	+	47.4(10.7)		

* Minus sign (−) denotes negative remote history of concussion (*n* = 60), plus sign (+) denotes remote history positive for at least one concussion (*n* = 33).

** Traditional neuropsychological test scores reported as age-corrected *T*-scores.

## PUBLIC INTEREST STATEMENT

Theoretical constructs are intangible and hypothetical variables used to help understand human behavior. When a new psychological test is developed to measure a theoretical construct, it is critical that the test have adequate *construct validity*, which means that the test indeed measures the construct of interest. *Convergent validity* refers to the extent to which a test measures the same thing as other tests purported to measure that construct (i.e. scores on all tests of verbal memory should be highly correlated) and *discriminant validity* describes the extent to which a test does not measure other, unrelated constructs (i.e. verbal memory test scores should not necessarily correlate with reaction time scores). This study investigated how healthy athlete’s performance on the ImPACT test battery compared to their performance on traditional neuropsychological tests whose construct validity is well understood. It was found that the construct validity of ImPACT subtests was variable leading to the conclusion that more research is needed prior to ImPACT being used as a clinical assessment.
